# Functional Outcomes and Quality of Life After Sacrum Fractures Managed by Either Operative or Conservative Approaches: A Case Series With One-Year Follow-Up

**DOI:** 10.7759/cureus.59375

**Published:** 2024-04-30

**Authors:** Anurag Baghel, Mohit K Verma, Pulak Sharma, Kumar Keshav, Amit Kumar, Sadhak Raghav

**Affiliations:** 1 Orthopaedics, Sanjay Gandhi Postgraduate Institute of Medical Sciences, Lucknow, IND; 2 Orthopaedics and Trauma, Sanjay Gandhi Postgraduate Institute of Medical Sciences, Lucknow, IND

**Keywords:** neurological dysfunction, functional outcome, sexual dysfunction, quality of life (qol), sacrum fracture

## Abstract

Background

Pelvic fractures caused by high-energy trauma, such as motor vehicle accidents or falls from a considerable height, commonly lead to sacral fractures. Approximately a quarter of sacral fractures are linked to neurological injury, and overlooking these fractures may result in neurological issues such as sexual dysfunction, hindered lower limb functionality, and urinary and rectal difficulties. The main goal of this study is to introduce our patient group who underwent either operative or nonoperative treatment for sacral fractures, with a follow-up period of one year, and assess their functional outcomes.

Methodology

This is a retrospective review of prospectively collected data from a consecutive series of patients at the Apex Trauma Centre, Sanjay Gandhi Postgraduate Institute of Medical Sciences, Lucknow. A consecutive series of 24 patients (17-55 years old) with sacral fractures treated either operatively or nonoperatively from 2018 to 2023 was studied. A total of 20 patients were available for follow-up questionnaires, and 20 patients participated in a physical examination. Time to final follow-up averaged 27.19 months (range = 12-57 months). The personal data of each patient was collected, including gender, age, comorbidities, concomitant injuries, mechanism of injury, fracture pattern/classification, surgical or nonsurgical treatment, other surgeries, length of surgery, length of hospital stays, adverse events, complications, neurologic and/or motor deficits, bowel and bladder function, and mortality. At a minimum one-year follow-up, the Majeed score, Oswestry Disability Index (ODI) questionnaire, and Gibbon’s classification were assessed.

Results

All fractures were healed. Five patients showed neurological weakness, with three patients having only paresthesia and two patients having lower limb weakness. The mean Majeed score was 75.4, representing a moderate clinical outcome. Final ODI scores averaged 10.6, representing mild disability among patients with sacrum fractures. Overall, 40% of sacrum fractures were associated with sexual dysfunction, with 30% of females and 50% of males reporting this issue. There was no significant difference (p > 0.05) between operated and conservatively managed sacrum fractures concerning ODI scores, neurological deficit, and sexual dysfunction.

Conclusions

Both male and female patients with traumatic sacrum fractures experienced a significant decrease in their quality of life and sexual function at least 12 months after their surgery. Sacrum fractures are associated with an increased prevalence of sexual dysfunction and bowel/bladder incontinence. Our study findings indicate that patients with sacrum fractures experience similar functional outcomes and incidences of sexual dysfunction irrespective of whether they are managed operatively or conservatively.

## Introduction

Pelvic fractures caused by high-energy trauma, such as motor vehicle accidents or falls from a considerable height, commonly lead to sacral fractures. Detecting and treating sacral fractures require a high degree of suspicion and clinical judgment for precise diagnosis and management [1]. Fractures are present in 30% to 45% of pelvic ring injuries, stemming from a range of causes, such as high-energy trauma to low-energy falls in osteoporotic individuals. It is important to highlight that 5% of sacral fractures have historically presented as isolated injuries [2]. The Denis, Roy-Camille (with Strange-Vognsen modification), and alphabet description are common classification systems for sacral fractures. However, the lack of consistency in displacement and comminution leads to a shortage of standardized reporting and classification of fracture morphology.

Sacral fractures present a diagnostic challenge due to their frequent lack of visibility on standard radiographs and potential overshadowing by other injuries in polytrauma patients. As a result, a significant proportion of sacral fractures, around 30%, are only detected at a later stage. Moreover, approximately a quarter of sacral fractures are linked to neurological injury, and overlooking these fractures may result in neurological issues such as sexual dysfunction, urinary and rectal difficulties, and hindered lower limb functionality [3,4].

Research on the conservative management of sacral fractures is primarily based on small case reports or subsets within larger studies, with restricted assessments of clinical results and lacking validated functional outcomes. Traditionally, nonoperative management has been the preferred method for treating these fractures, except in cases of pelvic ring instability. Although some recent studies suggest surgical intervention for sacral fractures, there is a lack of established criteria for surgical indications in this uncommon fracture type.

## Materials and methods

Ethical clearance was obtained from the Institutional Ethics Committee, Bioethics Cell, Sanjay Gandhi Postgraduate Institute of Medical Sciences, Lucknow (approval number: AN15-V4/SGSOP 03/V3). During a span of five years (2018-2023), a total of 24 patients with sacral fractures underwent either operative or nonoperative treatment. These patients were identified from a comprehensive retrospective analysis of the orthopedic trauma database and were subsequently invited for follow-up appointments at the outpatient department (OPD). Regrettably, four patients had passed away, while the remaining 20 patients willingly agreed to participate in the study.

The personal information of each patient was gathered, encompassing gender, age, comorbidities, concomitant injuries, mechanism of injury, fracture pattern/classification, surgical or nonsurgical treatment, other surgeries, length of surgery, length of hospital stay, adverse events, complications, neurologic and/or motor deficits, bowel and bladder function, and mortality. Patients were scheduled for follow-up appointments in the OPD at least one year post-injury, and data were obtained through questionnaires. Functional outcomes were assessed using the Majeed score, Oswestry Disability Index (ODI) questionnaire, and Gibbon’s classification. These functional outcomes were then categorized as follows: ≥85 as excellent, 70-84 as good, 55-69 as fair, and <55 as poor. No formal surgical protocol was implemented for sacral fractures.

We employed statistical methods to examine the relationships between various variables, utilizing descriptive statistics such as means, medians, and frequencies to summarize the collected data. Descriptive statistics were used to summarize patient demographics and fracture characteristics. An independent-sample t-test of association was used to compare the two sample means. The association was considered significant at p-values <0.05.

## Results

The study included 20 patients, with an equal distribution of 10 males and 10 females. The average age of the patients when they were injured was 29.25 years, ranging from 17 to 55 years. Motor vehicle accidents were the most common cause of trauma, accounting for 70% of the cases (14 out of 20), followed by falls from a height in six (30%) cases (Table 1). Among the associated injuries, acetabular fractures were present in six (30%) cases, while neurological deficits according to the American Spinal Injury Association score were observed in five (25%) cases. Gibbon’s classification identified three patients with paresthesia and two patients with lower limb weakness (Table 2). Additionally, six (30%) patients had abdominal injuries, including two cases of bladder neck tear, two cases of perineal tear, and two cases of blunt trauma to the abdomen (Table 1). Three (15%) patients had vertebral fractures, including one with a chance fracture of L1 along with transverse process fractures of L2 and L3, one with a burst fracture of L1 vertebrae, and one with a compression fracture of L1 vertebrae. All spine injuries were stable and managed nonoperatively (Table 1).

**Table 1:** Characteristics of the patients and surgery methods. M = male; F = female; RTA = road traffic accident; SI = sacroiliac

Patient	Sex	Age in years	Follow-up in months	Associated injury	Mechanism	Dennis classification	Treatment
1	M	34	12.40	None	RTA	1	Conservative
2	M	46	22.47	Bladder neck tear	Fall from a height	1	SI screw + symphyseal plating
3	M	21	19.43	Bladder neck tear	RTA	1	SI screw + symphyseal plating
4	F	27	13.83	Pubic rami	Fall from a height	3	Pelvic ex-fix
5	M	18	47.93	Pelvic	RTA	2	Pelvic ex-fix
6	M	34	25.80	Pubic rami	RTA	1	Conservative
7	M	38	13.43	None	RTA	1	SI screw + symphyseal plating
8	M	36	54.60	Perineal tear	RTA	1	Operative (SI screw)
9	F	20	19.73	Pubic rami and L2-L5 vertebrae	Fall from a height	2	SI screw fixation
10	F	25	56.60	TP of L2 L3	Fall from a height	1	Pelvic ex-fix
11	M	22	10.23	Blunt trauma to the abdomen	RTA	1	Conservative
12	M	20	12.83	Pubic rami	RTA	1	Spinopelvic fixation
13	F	35	17.00	Blunt trauma to the abdomen	RTA	1	Conservative
14	F	19	14.87	Vaginal tear	RTA	1	SI screw fixation
15	F	32	13.93	None	Fall from a height	2	SI screw
16	M	28	17.73	None	RTA	1	Conservative
17	F	19	13.77	Pelvic ring	RTA	1	Conservative
18	F	55	50.73	Pubic rami	Fall from a height	1	SI screw
19	F	17	57.07	Burst L1 vertebrae	RTA	1	Conservative
20	F	25	49.47	B/L acetabulum	RTA	1	Conservative

**Table 2:** Characteristics of the patients and functional scores. M = male; F = female

Patient	Sex	Age	Majeed score	Oswestry Disability Index	Gibbon’s classification	Sexual dysfunction
1	M	34	79	11	1	Absent
2	M	46	84	0	1	Absent
3	M	21	79	5	1	Present
4	F	27	69	17	1	Present
5	M	18	55	0	3	Absent
6	M	34	78	23	1	Present
7	M	38	63	10	1	Present
8	M	36	83	1	1	Present
9	F	20	81	14	1	Present
10	F	25	84	0	1	Absent
11	M	22	66	10	1	Absent
12	M	20	57	19	2	Present
13	F	35	72	25	3	Absent
14	F	19	70	29	1	Present
15	F	32	84	1	1	Absent
16	M	28	72	3	1	Absent
17	F	19	81	33	2	Absent
18	F	55	84	0	1	Absent
19	F	17	79	8	2	Present
20	F	25	88	3	1	Absent

Each patient underwent pelvic CT to obtain a more precise diagnosis and visualization of the fracture pattern. CT images were used to classify sacral fractures according to the Roy-Camille and Denis classifications. Among the 20 patients, 16 (80%) had zone 1 injuries, three (15%) had zone 2 injuries, and one (5%) had a zone 3 sacral fracture (Table 1). The choice of fracture fixation, surgical approaches, and techniques depended on factors such as fracture displacement, pelvic ring stability, injuries to other visceral organs, and the preference of the attending surgeon. Overall, 40% (eight patients) of sacrum fractures were found to be associated with sexual dysfunction, with 30% (three patients) of females and 50% (five patients) of males reporting this issue (Table 2). All surgeries were performed by the same team of experienced orthopedic surgeons.

**Figure 1:**
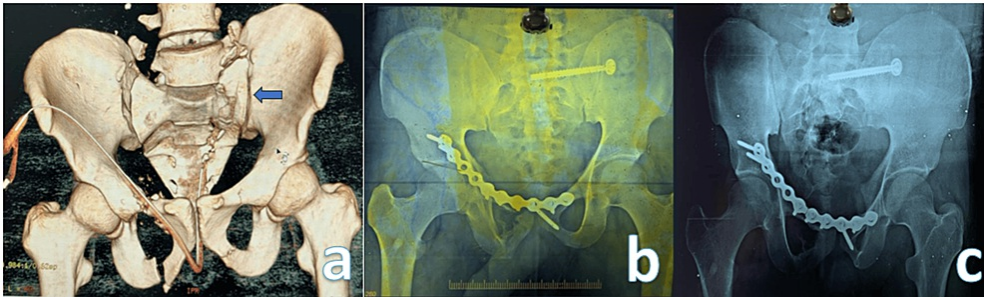
(A, B) Sacrum fracture fixation using a sacroiliac screw. (C) One-year follow-up X-ray.

**Figure 2:**
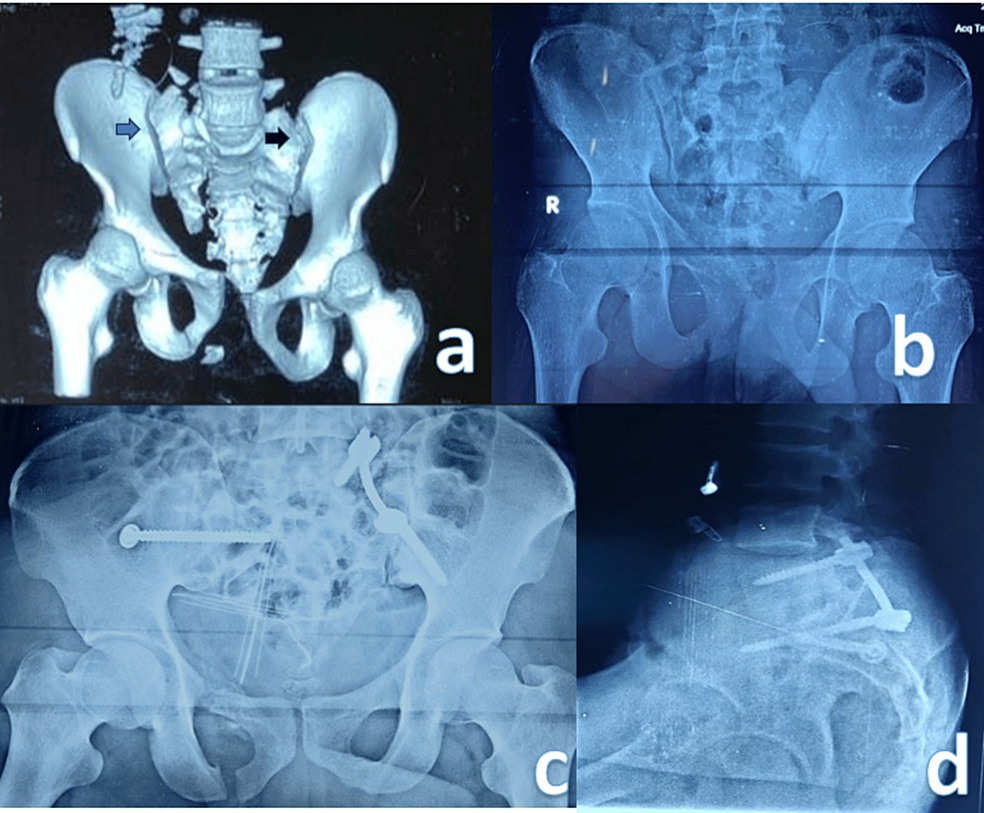
(A-D) Sacrum fracture fixation using spinopelvic fixation and transverse sacral alar fracture fixation using a sacroiliac screw.

**Figure 3:**
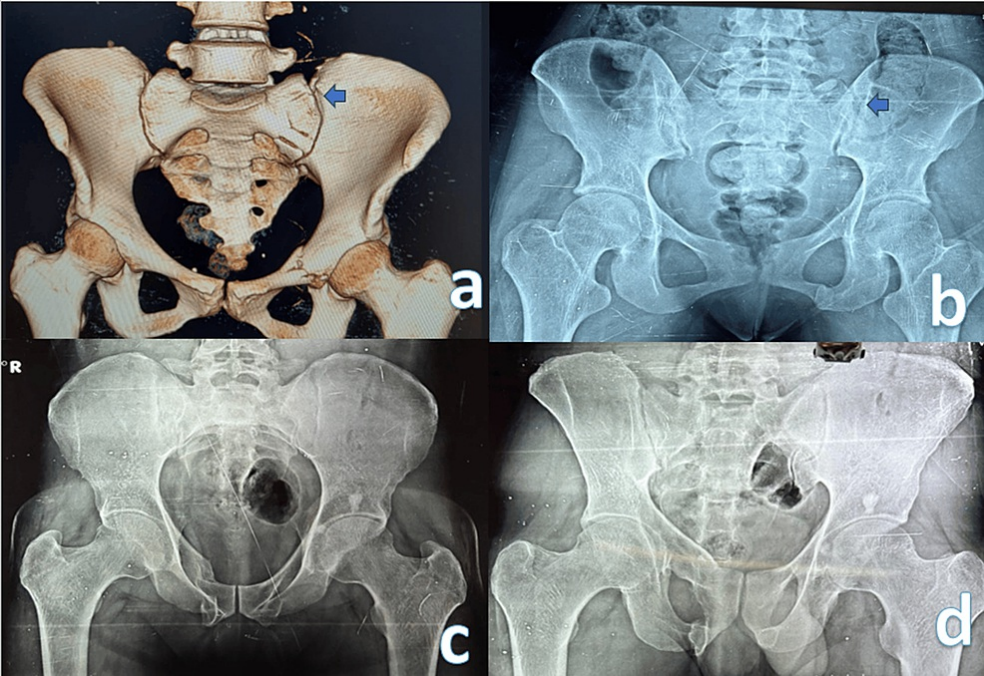
(A, B) Sacrum fracture Dennis type 1 managed conservatively. (C, D) One-year follow-up X-ray.

Closed reduction-internal fixation was the treatment of choice in the majority of cases. The sacral screw fixation (Figure 1) was the most commonly used fixation type, accounting for eight (40%) cases. In three cases, symphyseal plating was performed along with sacroiliac screw fixation to address pubic symphysis diastasis. For patients with severe urinary bladder injury, a combination of anterior definitive external fixation and percutaneous screw fixation of the posterior pelvic ring was employed. In one patient with a Dennis zone 3 injury, spinopelvic fixation in the form of triangular fixation was performed (Figure 2). Conservative management was opted for in eight patients (Figure 3).

Among the 20 cases of sacrum fractures, 12 were treated operatively using different surgical techniques. The mean ODI was 8 for operated patients and 14.5 for those managed conservatively, indicating a higher level of disability in the latter group. Additionally, patients without neurological deficits were more common in the nonoperative group, while sexual dysfunction was more prevalent among those who underwent surgery (Table 3). This can be attributed to a higher incidence of sexual dysfunction in Dennis type 2 and 3 fracture patterns which necessitated operative management. An independent-sample t-test was performed to determine if there were differences in ODI scores, neurological deficit, and occurrence of sexual dysfunction between operated and conservative treatment. There was no significant difference (p > 0.05) between operated and conservatively managed sacrum fractures concerning ODI scores, neurological deficit, and occurrence of sexual dysfunction (Table 3).

**Table 3:** Comparison of Oswestry Disability Index, neurological deficits, and sexual dysfunction between operative and conservative management using an independent-sample t-test.

Treatment	Oswestry Disability Index		Neurological deficit		Sexual dysfunction	
	Operative (N = 12)	Conservative (N = 8)	Operative (N = 12)	Conservative (N = 8)	Operative (N = 12)	Conservative (N = 8)
Mean	8	14.5	1.25	1.5	0.5	0.375
SD	9.75	11.11	0.62	0.76	0.52	0.52
P-value	0.184		0.451		0.606	

## Discussion

Our research found that every patient with a sacrum fracture experienced high-energy trauma, with motor vehicle accidents being the leading cause at 70%, followed by falls from a height at 30%. The predominant mechanism of sacrum fracture trauma, as indicated by most studies, aligns with our findings of motor vehicle accidents [5,6].

The goal of managing these injuries is to quickly perform definitive stabilization to reduce pain, minimize the use of pain medication, and decrease the risk of complications, such as deep vein thrombosis, pulmonary thromboembolisms, and pressure ulcers, by promoting faster patient mobility [7,8]. According to the most recent meta-analysis by Bäcker et al., 68.1% of individuals who suffer from U-shaped sacral fractures develop neurological lesions. Furthermore, the study also revealed that 65.1% of these patients show signs of recovery following surgical intervention [9]. According to our study, 25% of all fractures were associated with neurological impairment.

In a comprehensive review of 236 sacrum fracture cases by Denis et al. [2], a particular treatment approach was highlighted, focusing on addressing neurological issues to facilitate functional recuperation once the evident pelvic trauma injury had fully healed. In a multicentric observational study by Wright et al. [10], certain fracture patterns were indicative of sexual and excretory dysfunction in both men and women one year following a pelvic fracture. Overall, 21% of patients with pelvic fractures experienced sexual dysfunction compared to 14% of those without pelvic fractures. Additionally, 8% of patients with pelvic fractures reported bowel or bladder incontinence, while only 4% of those without pelvic fractures reported the same. Men with sacroiliac fractures and women with symphyseal diastasis are more likely to experience these dysfunctions. Additionally, individuals with sacrum fractures, particularly men with sexual dysfunction and women with excretory dysfunction, tend to have a notably lower quality of life.

In their study, Harvey-Kelly et al. [11] demonstrated the impact of sexual dysfunction on quality of life following traumatic pelvic fractures. They found that 43.8% of female and 52.1% of male patients experienced sexual dysfunction, which was identified as an independent risk factor for decreased quality of life post-injury. Our study revealed that 40% of sacrum fractures were associated with sexual dysfunction, affecting a significant number of patients, with 30% of females and 50% of males reporting this issue. Both male and female patients with traumatic pelvic fractures experienced a notable decline in quality of life and sexual function at least one year after their surgery.

In a comprehensive literature review, Walton et al. [12] documented the occurrence of female sexual dysfunction after pelvic fracture. The prevalence of female sexual dysfunction following pelvic fracture ranged from 25% to 62%. The most frequently reported issues included challenges during intercourse, dyspareunia, orgasmic dysfunction, genitourinary pain, reduced interest in intercourse, decreased satisfaction with intercourse, and pelvic floor dysfunction.

In a retrospective case series, Copuroglu et al. [13] reported that 45% of individuals with pelvic fractures exhibited signs of potential sexual dysfunction based on their Arizona Sexual Experience Scale scores, with 10% requiring psychiatric assessment. This study underscored the significance of addressing and managing sexual issues in this patient population. Rodrigues-Pinto et al. [4] demonstrated that isolated sacral fractures account for only 5% of cases, while up to 45% of sacral fractures are accompanied by a pelvic ring injury. Additionally, the presence of associated injuries frequently plays a crucial role in determining the prognosis of patients with sacral fractures [4]. Our study revealed that 80% of sacrum fractures were accompanied by additional injuries. Our findings showed that 30% of these cases involved abdominal injuries, including bladder neck tears. Furthermore, 30% of the cases also involved simultaneous acetabular and pelvic ring injuries, while 20% exhibited vertebral fractures.

The majority of patients in our study underwent indirect decompression; however, we did not observe any significant differences in functional outcomes, quality of life, or time to surgery. Kepler et al. [14] conducted a literature review and similarly discovered that there was no distinction in neurological recovery between direct and indirect decompression techniques. Assessing functional outcomes and quality of life of patients with sacrum fractures is a complex task due to the rarity of these fractures. Moreover, the small number of patients who experience such fractures often face additional injuries that further impact their overall outcomes.

Lindahl et al. [15] reported that full displacement of the transverse sacral fracture, along with paraplegia or paraparesis, were indicators of unfavorable clinical prognosis. Ruatti et al. [16] examined functional results following U-shaped fractures by utilizing the Majeed and Iowa surveys. In contrast to our findings, which showed a mean Majeed score of 75.4 points, improved outcomes were achieved in the Ruatti et al. study. However, it is important to note that the patients in their study had isolated sacral fractures, which could have potentially influenced their higher results. The average duration of the follow-up after surgery was 27.19 months. Tian et al. [17]. found that the functional outcomes assessed using a Majeed questionnaire were excellent for 12 (66%) patients, good for four (22%) patients, and fair for two (11%) patients.

Hu et al. [18] also found that 13 (59%) patients had excellent functional outcomes, while six (27%) had good outcomes, two (9%) had fair outcomes, and one (4%) had poor outcomes. Triangular osteosynthesis represents a recent advancement in treating vertical unstable sacral fractures. This type of fixation is characterized by its rigidity, allowing for early full weight-bearing, thus providing better functional outcomes [19]. Jindal et al. [20] discovered that triangular fixation in unstable transforaminal sacral fractures offers dependable stability and expedited functional recovery. The objective of triangular fixation in these fractures is to achieve proper alignment and adequate stability to counteract translational and rotational forces. Triangular osteosynthesis combines transverse fixation with lumbopelvic osteosynthesis, resulting in strong stability in multiple planes [20]. Additionally, nerve decompression can be easily performed, ultimately aiding in the recovery of function. He et al. [21] in their study found that surgical intervention led to enhanced neurological function in the lower extremities of patients with U-shaped sacral fractures. Nevertheless, there was no improvement in bowel and sexual functions, and urinary function worsened as time progressed. It appears that most impairments become permanent if they persist for one year post-surgery [21].

Our study has the potential to encourage further randomized comparative studies. Moreover, we believe that the presence of concomitant injuries, complications, and more severe initial injuries had a significant impact on the suboptimal performance in patient outcomes. It is worth noting that there were no significant differences in the therapy provided to these patients. However, there are a few limitations to consider in this study. First, it was conducted at a single center, which may limit the generalizability of the findings. Second, there was no standardized surgery protocol for sacrum fracture fixation, as the choice of technique and fixation depended on the attending surgeon’s preference. Additionally, due to the heterogeneity of associated injuries and the small sample size, it was not possible to determine the impact of other injuries on quality of life and functional outcomes.

## Conclusions

Both male and female patients with traumatic sacrum fractures experienced a significant decrease in their quality of life and sexual function at least 12 months after their surgery. Our study findings indicate that patients with sacrum fractures experience similar functional outcomes and incidences of sexual dysfunction irrespective of whether they are managed operatively or conservatively. This emphasizes the importance of conducting additional research on functional outcomes and sexual dysfunction in patients with sacrum fractures. Nonetheless, considering the rarity of this trauma, our study stands out as one of the few that presents functional outcomes and quality of life after sacrum fracture. Therefore, our findings can serve as a valuable reference for future studies.
